# Structural and functional role of Domain I for the insecticidal activity of the Vip3Aa protein from *Bacillus thuringiensis*

**DOI:** 10.1111/1751-7915.14110

**Published:** 2022-07-13

**Authors:** Maria Lázaro‐Berenguer, Francisco Paredes‐Martínez, Yolanda Bel, Rafael Núñez‐Ramírez, Ernesto Arias‐Palomo, Patricia Casino, Juan Ferré

**Affiliations:** ^1^ Department of Genetics Universitat de València Burjassot Spain; ^2^ Institut Universitari de Biotecnologia i Biomedicina BIOTECMED Universitat de València Burjassot Spain; ^3^ Department of Biochemistry and Molecular Biology Universitat de València Burjassot Spain; ^4^ Centro de Investigaciones Biológicas Margarita Salas CSIC Madrid Spain; ^5^ CIBER de Enfermedades Raras (CIBERER‐ISCIII) Madrid Spain

## Abstract

Vip3 proteins are produced by *Bacillus thuringiensis* and are toxic against lepidopterans, reason why the *vip3Aa* gene has been introduced into cotton and corn to control agricultural pests. Recently, the structure of Vip3 proteins has been determined and consists of a tetramer where each monomer is composed of five structural domains. The transition from protoxin to the trypsin‐activated form involves a major conformational change of the *N*‐terminal Domain I, which is remodelled into a tetrameric coiled‐coil structure that is thought to insert into the apical membrane of the midgut cells. To better understand the relevance of this major change in Domain I for the insecticidal activity, we have generated several mutants aimed to alter the activity and remodelling capacity of this central region to understand its function. These mutants have been characterized by proteolytic processing, negative staining electron microscopy, and toxicity bioassays against *Spodoptera exigua.* The results show the crucial role of helix α1 for the insecticidal activity and in restraining the Domain I in the protoxin conformation, the importance of the remodelling of helices α2 and α3, the proteolytic processing that takes place between Domains I and II, and the role of the C‐t Domains IV and V to sustain the conformational change necessary for toxicity.

## Graphical abstract

To better understand the relevance of the major change in Domain I of Vip3 proteins in the transition from protoxin to activated toxin for the insecticidal activity, we have generated several Vip3Aa mutants aimed to alter the activity and remodelling capacity of this central region to understand its function. These mutants have been characterized by proteolytic processing, negative staining electron microscopy, and toxicity bioassays against *Spodoptera exigua*. The results show the crucial role of helix α1 for the insecticidal activity and for restraining the Domain I in the protoxin conformation, the importance of the remodelling of helices α2 and α3, the proteolytic processing that takes place between Domains I and II, and the role of the C‐t Domains IV and V to sustain the conformational change necessary for toxicity.

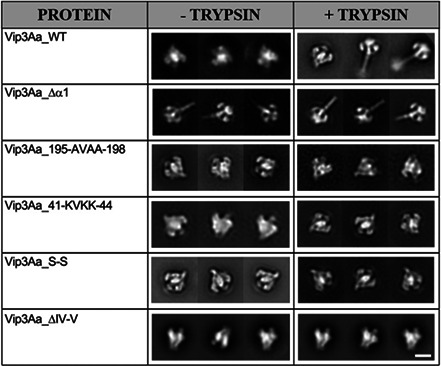

## INTRODUCTION

Vegetative insecticidal proteins (Vip3) are produced during the vegetative growth phase of the bacterium *Bacillus thuringiensis*, and are toxic against a wide range of lepidopteran pests (Chakroun et al., 2016; Estruch et al., 1996; Palma et al., 2014; Ruiz de Escudero et al., 2014). Notably, members of the Vip3A family have been used in Bt crops (crops that express one or more *B. thuringiensis* genes) in combination with the Cry insecticidal proteins to prevent the evolution of insect‐resistant populations (Carrière et al., 2015; Tabashnik & Carrière, 2017), since they are toxic for insect species that have already developed resistance against the Cry proteins and do not share binding sites with them (Chakroun & Ferré, 2014; Gouffon et al., 2011; Lee et al., 2006; Quan et al., 2021; Sena et al., 2009).

The Vip3 proteins are secreted as protoxins of ~89 kDa which, once ingested by the larvae, are processed by the midgut proteases into two different fragments of 19–22 and 62–66 kDa that remain attached and conform to the activated protein (Bel et al., 2017; Chakroun & Ferré, 2014; Quan & Ferré, 2019; Zack et al., 2017). Once processed, Vip3 proteins are able to bind to specific receptors located on the brush border membrane of the midgut epithelium cells (Chakroun & Ferré, 2014; Quan et al., 2021) where they exert their toxic effect. Regarding their mode of action, it has been shown that Vip3 proteins get inserted into the membrane and induce pore formation, leading to the death of the larvae (Lee et al., 2003; Liu et al., 2011). In addition, and mainly in studies with culture cells, it has been reported that Vip3A proteins can induce apoptosis through the mitochondrial apoptotic pathway after being internalized into the cells (Hernández‐Martínez et al., 2017; Hou et al., 2020; Jiang et al., 2016; Nimsanor et al., 2020).

**FIGURE 1:**
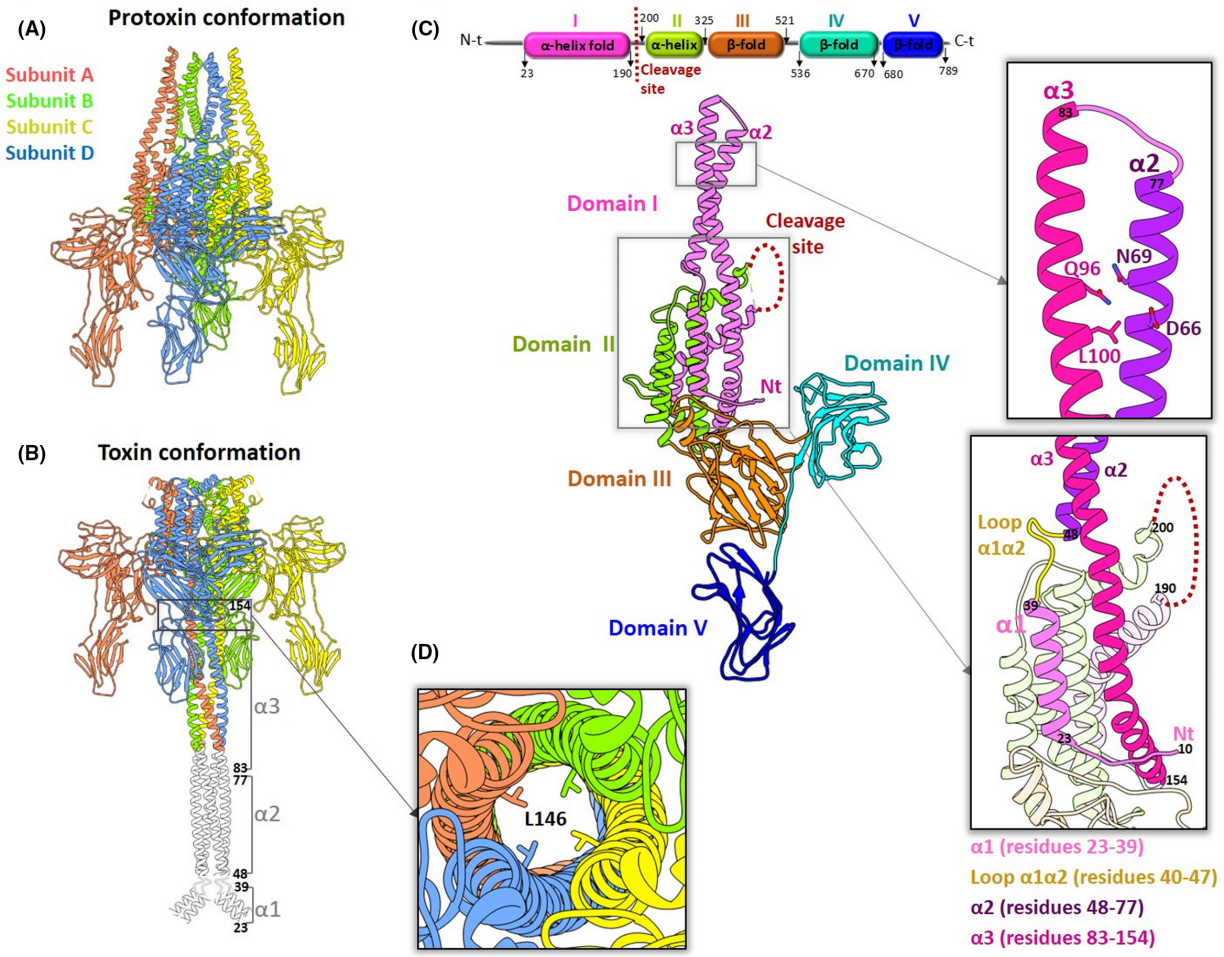
Structural features of Vip3Aa. (A) Protoxin conformation of Vip3Aa (PDB: 6TFJ; subunits in the tetramer are coloured coded). (B) Toxin conformation of Vip3Aa (PDB: 6TFK) composed by a tetrameric coiled‐coil traced till residue 95. Modelled coiled‐coil in white till residue 23. Helices forming the coiled‐coil are labelled and numbered. (C) Domain composition of a subunit from the protoxin conformation highlighting each domain coloured coded and the loop containing the cleavage site. Boxes are extracted to zoom the helices α2 and α3 with the residues mutated to Cys for disulphide bond cross‐linking and to zoom helix α1 connected to helix α2 by loop α1α2. (D) Zoom of a box shown in B showing the internal tetrameric coiled‐coil with L146 coming from each subunit.

Recently, the structure of Vip3 proteins has been determined by cryo‐electron microscopy (cryo‐EM), revealing that they consist of a homotetramer in which each subunit is composed of five structural domains (Byrne et al., 2021; Núñez‐Ramírez et al., 2020; Zheng et al., 2020) (Figure 1). Comparison between Vip3 protoxin and trypsin‐activated protein structures has demonstrated a major conformational change at the N‐terminal (N‐t) Domain I (Figure 1A, B). In the protoxin conformation, the N‐t forms a helix bundle that is remodelled into a tetrameric coiled‐coil as observed in the toxin conformation, with a flexible end that most probably is formed by helix α1 (Byrne et al., 2021; Núñez‐Ramírez et al., 2020). The tip of this coiled‐coil structure has been shown to get inserted into the membrane of liposomes (Byrne et al., 2021). At the C‐terminus (C‐t), Domains IV and V (Figure 1C) are carbohydrate‐binding motifs that do not contact the N‐t part of the protein or directly interact with other subunits, and are dispensable for oligomer formation (Byrne et al., 2021; Núñez‐Ramírez et al., 2020; Quan & Ferré, 2019; Zheng et al., 2020). Also, it has recently been shown that they are not necessary for the specific binding of Vip3Af to *Spodoptera* spp. brush border membrane vesicles (BBMV) and *Spodoptera frugiperda* Sf21 cultured cells, or for exerting toxicity against the latter (Jiang et al., 2020; Quan et al., 2021). Nevertheless, many reports have shown that they are necessary for the toxicity of Vip3A proteins in vivo (Banyuls et al., 2018, 2021; Gayen et al., 2012, 2015; Li et al., 2007; Quan & Ferré, 2019; Selvapandiyan et al., 2001).

To better understand the relevance of Domain I for the toxicity and the role of the C‐t Domains IV and V in the in vivo toxicity of Vip3Aa, we have engineered a deletion mutant of these domains in addition to mutations aimed to restrict remodelling of Domain I as well as to modify it. Since activation of Vip3A proteins by the insect midgut proteases is a key step for the insecticidal activity (Chakroun et al., 2016; Hernández‐Martínez et al., 2013; Lee et al., 2003), the behavior of these mutants upon proteolytic processing has been characterized. Also, we have performed negative staining EM and toxicity bioassays against the lepidopteran species *Spodoptera exigua*, to determine the effect of these mutations on the protein structure and how this affects the insecticidal activity. The results demonstrate that the integrity and the remodelling of Domain I with subsequent coiled‐coil formation is essential for the in vivo insecticidal activity of Vip3Aa.

## EXPERIMENTAL PROCEDURES

### Mutagenesis of Vip3Aa

Vip3Aa16 (NCBI accession No. AAW65132) (residues 10–789) was cloned in LIC 1.5, as previously described (Núñez‐Ramírez et al., 2020), and then was used as a template vector to prepare the mutant vectors used in this study. Specifically, the sequences of Vip3Aa_∆α1 and Vip3Aa_∆IV‐V were prepared by amplification of the vector template from residue 40 to 789 and residue 10 to 535, respectively, then cloned in LIC 1.5. The rest of the mutants were obtained using the Q5 Site‐Directed Mutagenesis Kit (New England Biolabs) with the vector template to obtain the corresponding mutant vectors containing the mutant proteins. See Table S1 for the primer description used to prepare each mutant protein.

### Protein expression and purification

The production of Vip3Aa WT and mutants used in this study was described previously (Núñez‐Ramírez et al., 2020). Briefly, protein expression was conducted in *Escherichia coli* C43 (DE3) containing each of the mutant vectors. Cells were grown in Luria‐Bertani broth to exponential phase (OD_600_ ~ 0.6), then induced with 0.5 mM isopropyl β‐d‐1‐thiogalactopyranoside (IPTG), incubated at 37°C for 3 h and then centrifuged and stored at −20°C. For purification, thawed cells were resuspended in buffer A (50 mM Tris–HCl, 0.5 M NaCl, and 50 mM MgCl_2_) pH 8.0, with 1 mM phenylmethanesulfonyl fluoride and 1 mM Tris (2‐carboxyethyl)phosphine hydrochloride, and sonicated for 5 min. The cell lysate was centrifuged at 25,000 *g* for 30 min and the clarified supernatant was loaded into a 5 ml Streptrap column (GE, Healthcare). The protein was eluted with 5 mM *d*‐desthiobiotin dissolved in buffer A pH 8.0. The StrepII‐tag was removed adding 3C PreScission protease (fused to an N‐terminal GST‐tag) to the Vip3Aa mutants at a 1:1/25 (protein:protease) molar ratio followed by dialysis. The sample was then subjected to two other steps of affinity chromatography with a Streptrap and a GST‐Trap column to separate the non‐tagged protein from any remaining tagged protein and protease. The non‐tagged protein was further purified by gel filtration chromatography using a ProteoSEC 16/60 6–600 HR (Generon, UK). Samples containing the purest protein were concentrated to ~5 mg/ml, aliquoted, frozen with liquid nitrogen, and stored at −80°C for further use.

### In vitro proteolytic assays

Purified proteins diluted in buffer A pH 7.5 were quantified by Bradford (1976). A fixed amount of protein (10 μg) was incubated with 10% (w/w; 1 μg) commercial trypsin or α‐chymotrypsin (both from bovine pancreas, Sigma Aldrich), or 10% (w/w) midgut juice or 10% (w/w) BBMV from *S. exigua*. Midgut juice of *S. exigua* was prepared following the protocol described elsewhere (Chakroun et al., 2012), flash‐frozen in liquid nitrogen and stored at −80°C. BBMV from *S. exigua* were prepared by the differential magnesium precipitation method (Wolfersberger, 1993) and stored at −80°C as well. The total protein concentration in the midgut juice and BBMV preparations was determined by the Bradford method just before use.

The reaction was carried out in a final volume of 20 μl, in buffer A pH 7.5 or in 20 mM carbonate buffer pH 10, depending on the pH conditions required. All samples were incubated for 1.5 h at 37°C for trypsin and α‐chymotrypsin digestions and at 30°C for midgut juice and BBMV digestions. Then, samples were clarified by centrifugation (16,100 *g* for 10 min at 4°C) and the supernatant containing the processed proteins was incubated for 10 min at room temperature with 10 mM AEBSF [4‐(2‐aminoethyl)benzenesulfonyl fluoride HCl] serine protease inhibitor to avoid further processing of the proteins. Proteolytic fragments were separated on 12% SDS‐PAGE.

### Protein structure visualization by negative staining electron microscopy

Purified samples were analysed by electron microscopy after being adsorbed to glow‐discharged carbon‐coated grids and stained with 2% (w/v) uranyl acetate as described previously (Núñez‐Ramírez et al., 2020). Grids were observed using a JEOL JEM‐1230 transmission electron microscope, operated at 100 kV, at a nominal magnification of 40,000. EM images were taken under low dose conditions with a CMOS Tvips TemCam‐F416 camera at 2.84 Å per pixel.

Image processing was performed using the SCIPION package (de la Rosa‐Trevín et al., 2016). The contrast transfer function of the microscope for each micrograph was estimated using CTFFIND4 (Rohou & Grigorieff, 2015). Particles were then automatically picked using the Xmipp routine inside SCIPION (Abrishami et al., 2013) and subjected to 2D classification using RELION (Scheres, 2012).

### Insect rearing and toxicity bioassays

Surface contamination bioassays were performed with a laboratory population of *S. exigua* maintained on a semi‐synthetic diet (Bell & Joachim, 1976) in a rearing chamber at 25 ± 2°C, 70% ± 5% relative humidity, and 16:8 h light:dark. Increasing concentrations of purified proteins dissolved in buffer A pH 7.5, and quantified by the Bradford assay just before use, were prepared and dispensed (50 μl) over the surface of 2 cm^2^ wells filled with a semi‐synthetic diet; at least 16 wells were used for each protein concentration. After the diet surface was dry, one neonate larvae was gently placed into each well and the plates were sealed. Trays were maintained in a climatic chamber under the same conditions used for colony rearing and the mortality was recorded after 10 days of incubation. At least three biological replicates of the assay were performed for each protein. The toxicity of the proteins was quantified by Probit analysis using PoloPlus software. Graphical representations (Figure S1) were performed with GraphPad Prism version 5.

### In vivo proteolytic processing with *S. exigua* larvae and Western blot

Purified WT and Vip3Aa_195‐AVAA‐198 proteins, diluted in buffer A pH 7.5, were mixed with phenol red and sucrose (18 μl of 2 mg/ml protein +2 μl of phenol red +2.5 μl of 1.6 M Sucrose). Then, *S. exigua* L3 larvae were starved for at least 4 h and fed by drop‐feeding with the protein mixtures. Larvae that ingested the protein mixtures were separated and dissected at time intervals (15 min and 16 h post‐ingestion). Control larvae were just fed with the buffer‐dye mixture. Individual midguts of treated larvae were homogenized in 10 μl of 10 mM AEBSF and incubated at room temperature for at least 10 min to stop any further proteolytic processing of the proteins. Samples were subjected to 12% SDS‐PAGE along with a Dual Color Molecular Marker (Bio‐Rad). After SDS‐PAGE, the proteins were transferred to a nitrocellulose membrane (Ge healthcare Armershan™ Hybond™—ECL) by humid transference in blotting buffer (39 mM glycine, 48 mM Tris‐base, 0.037% SDS, 20% methanol) using the Bio‐Rad system. The lane of the molecular marker was separated from the rest using a clean razor. The two pieces of the membrane were blocked overnight with 5% skimmed milk dissolved in PBST buffer (1× PBS + 0.1% Tween 20). The part of the membrane containing the proteins was incubated for 1 h at room temperature (with shaking) with a primary antibody anti‐Vip3Aa16 raised in the rabbit at 1/5000 antibody dilution in PBST containing 0.5% skimmed milk. After 3 washing steps with PBST (5 min each), the membrane was incubated for 1 h at room temperature with the secondary antibody anti‐rabbit conjugated with HRP peroxidase (Sigma Aldrich) at 1/20.000 antibody dilution in PBST containing 0.5% skimmed milk. The part of the membrane containing the molecular marker was incubated for 1 h at room temperature with Precision Protein StrepTactin‐HRP (Bio‐Rad) at 1/20.000 dilution in PBST. After three washing steps (5 min each) with PBST, the bands were visualized with the Amersham ECL Prime Western Blotting Detection Reagent using Image Quant LAS 4000.

## RESULTS

### Engineering of Vip3Aa mutant proteins

To better understand the role of the structural features of Domain I in the toxicity, we have engineered several mutants targeting different regions or processes which can be grouped into four categories: (i) mutants directed to affect the N‐t part of the protein, named as Vip3Aa_∆α1, Vip3Aa_41‐KVKK‐44 and Vip3Aa_M34L, (ii) mutants directed to affect the proteolytic processing and conformational change of the activated protein, named as Vip3Aa_195‐AVAA‐198 and Vip3Aa_S‐S, (iii) mutants directed to reduce the internal diameter of the tetrameric coiled‐coil observed in the toxin conformation, named as Vip3Aa_L146F and Vip3Aa_L146M, and (iv) a mutant lacking the C‐t Domains IV and V (Vip3Aa_∆IV‐V). A detailed description of the mutations and their expected effect on the structure and/or properties of the protein, according to the structural data, are summarized in Table 1.

**TABLE 1:** Nomenclature and description of the Vip3Aa mutants

Category	Mutation name	Mutation description^*a*^	Expected consequence of the mutation
Mutants directed to affect the N‐t part of the protein and helix α1	Vip3Aa_∆α1	Deletion from N‐t to residue 39 which removes helix α1	To remove helix α1
	Vip3Aa_41‐KVKK‐44	Mutation of residues 41‐DTGG‐44 for 41‐KVKK‐44 that incorporates a new trypsin proteolytic cleavage site after helix α1	To insert a new trypsin cleavage site to remove helix α1
	Vip3Aa_M34L	Point mutation M34L in helix α1	To increase the insecticidal activity of the protein (Banyuls et al., 2021)
Mutants directed to affect the proteolytic processing and the conformational change of the activated protein	Vip3Aa_195‐AVAA‐198	Mutation of residues 195‐KVKK‐198 to 195‐AVAA‐198	The trypsin proteolytic cleavage site is changed to avoid trypsinization (Zhang et al., 2018)
	Vip3Aa_S‐S	Mutation to Cys of four residues located in domain I: D66C and N69C in helix α2 and Q96C and L100C in helix α3	To block the protoxin conformation by disulphide bridges formation through the introduced Cys residues
Mutants directed to reduce the inside diameter of the coiled‐coil	Vip3Aa_L146F	Mutation L146F in helix α3	To change the inside diameter of the coiled‐coil
	Vip3Aa_L146M	Mutation L146M in helix α3	To change the inside diameter of the coiled‐coil
Mutant directed to affect C‐t domains	Vip3Aa_∆DIV‐V	Deletion of C‐t domains IV and V	To check the role of C‐t domains in maintaining the structure and function

^a^ See Figure 1.

### In vitro proteolytic processing of the Vip3Aa WT and mutant proteins

**FIGURE 2:**
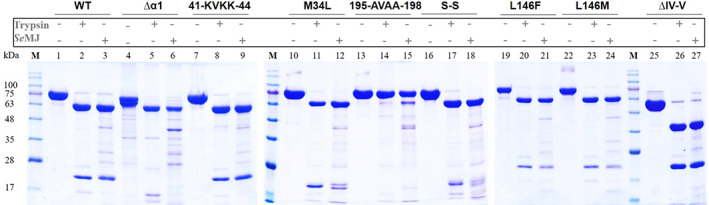
In vitro proteolytic processing of the Vip3Aa WT and mutant proteins. Proteins were incubated with either commercial trypsin (10% w/w at pH 7.5) or *S. exigua* midgut juice (10% w/w at pH 7.5) and the resulting fragments were separated in SDS‐PAGE. Band sizes were estimated according to the Blue Star molecular marker (M) (NIPPON Genetics).

The WT and the mutant proteins were subjected to proteolytic processing in vitro under two conditions: using commercial trypsin and using midgut juice of *S. exigua* larvae (*Se*MJ). As can be observed in Figure 2, the WT protein (~85 kDa), rendered two fragments of 20 and 65 kDa (corresponding to N‐t Domain I and C‐t Domains II–V, respectively) in both proteolytic conditions. The mutant Vip3Aa_Δα1, which lacks helix α1 (Figure 1C), showed a doubled band pattern before proteolysis (bands of ~80 and ~75 kDa) (Figure 2), probably due to different unfolding stages of the dissociated subunits under denaturing conditions affecting the electrophoretic mobility of protein–SDS interaction. MALDI‐TOF analysis of the two bands indicated that they were the same protein with 99.5% of confidence. Indeed, upon processing by trypsin, both bands disappeared rendering the 65 kDa band shown in the WT and an N‐t band that runs as ~16 kDa, instead of 20 kDa, due to the absence of helix α1. Using *Se*MJ, only the 65 kDa band was observed (Figure 2), presumably by the degradation of the ~16 kDa fragment by midgut proteases. The mutant Vip3Aa_41‐KVKK‐44, which includes a proteolytic site in the loop α1α2 aimed to remove helix α1 (Figure 1C), unexpectedly showed the same proteolytic pattern as the WT in both digestion conditions (Figure 2), indicating that this site was not sufficiently exposed to be cleaved by proteases. The mutant Vip3Aa_195‐AVAA‐198, designed to eliminate the trypsin proteolytic site KVKK located in the loop α4α5 between Domains I and II (Figure 1C), was not digested by the commercial trypsin, as previously described (Zhang et al., 2018). However, a slight band corresponding to the 65 kDa fragment was observed upon incubation with *Se*MJ, pointing to slight processing by the mixture of proteases in the midgut juice (Figure 2). The mutant Vip3Aa_S‐S, which contained mutations D66C and N69C located in helix α2, as well as, mutations Q96C and L100C located in helix α3, (Figure 1C) was designed to cross‐link helices α2‐α3 by two disulphide bonds (D66C‐L100C and N69C‐Q96C) to avoid remodelling of both helices upon activation. This mutant showed a similar proteolytic pattern to the WT protein under reducing conditions (Figure 2). However, under non‐reducing conditions the 20 kDa band runs faster in SDS‐PAGE (Figure S2) indicating a more compact conformation of Domain I probably due to the cross‐link between helices α2 and α3. The presence of the disulphide bonds was also confirmed by MALDI‐TOF (Figure S3) and LC–MS tandem MS (Figure S4).

The three single residue mutants, the mutant Vip3Aa_M34L, having a mutation within helix α1 (Figure 1C), and the mutants Vip3Aa_L146F and L146M designed to reduce the internal diameter of the tetrameric coiled‐coil upon activation (Figure 1D), as L146 is located in α3 (Figure 1B, C), all showed a similar pattern to the WT (Figure 2).

Finally, the mutant Vip3Aa_ΔIV‐V, lacking the C‐t Domains IV and V, showed a proteolytic pattern in both conditions composed of two bands, the 20 kDa for the Domain I and a ~38 kDa comprising Domains II and III (Figure 2).

Overall, all the mutants, except Vip3Aa_195‐AVAA‐198, showed proteolytic processing upon incubation with *Se*MJ or trypsin, although some of them had changes in the electrophoretic mobility of the protein bands compared to WT. The fact that Vip3Aa_195‐AVAA‐198 could not be processed by trypsin and slightly by *Se*MJ indicates that, in vivo, a different type of proteases may cleave the mutated binding site or there is an alternative cleavage site close to the mutated one.

### Negative staining—EM visualization of protoxin and trypsin‐treated Vip3Aa WT protein and its mutants

**FIGURE 3:**
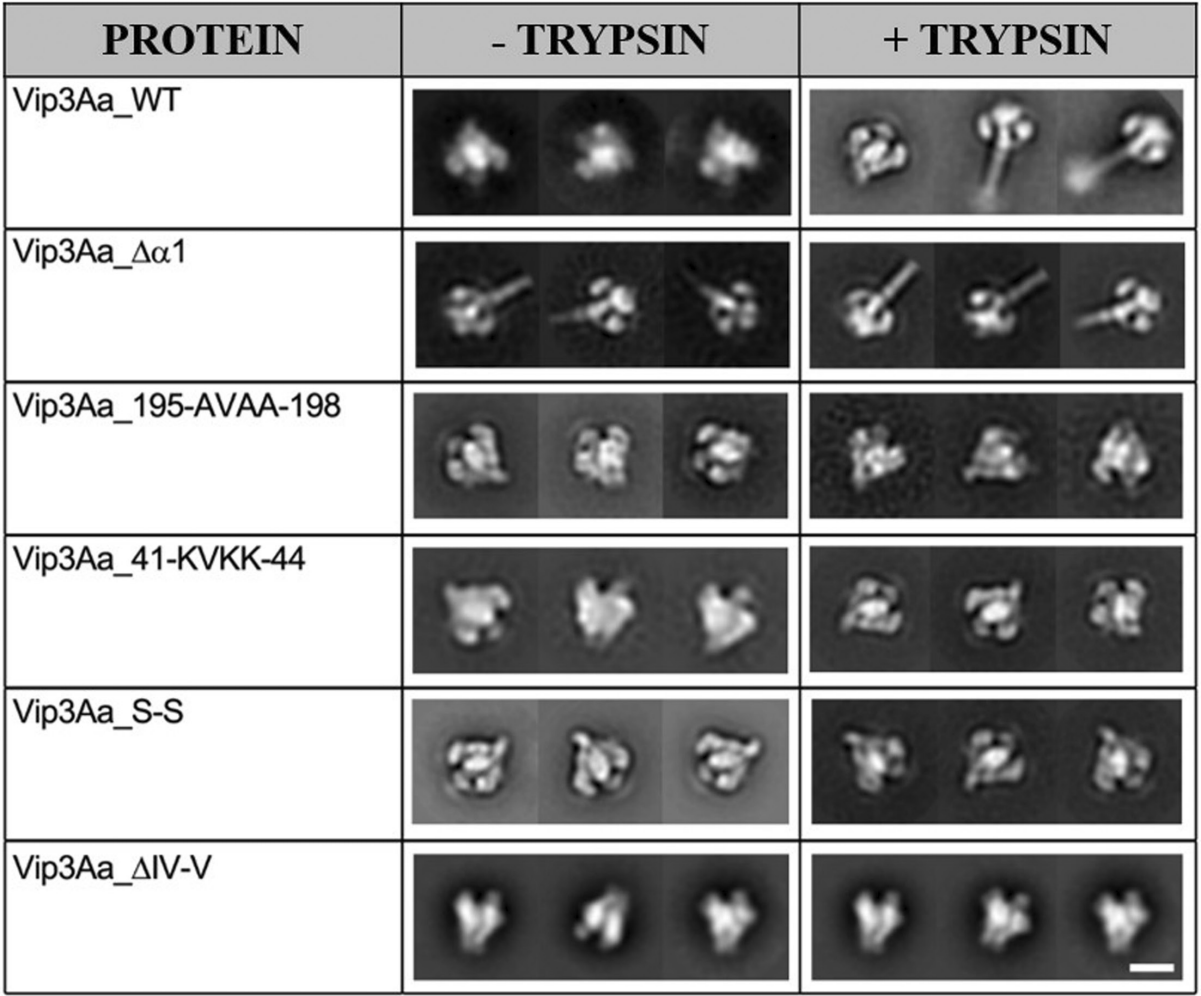
Negative staining transmission electron microscopy of Vip3Aa WT and selected mutants. Pictures were taken both in the protoxin form and after trypsin treatment of the samples. The bar at the bottom right represents 10 nm.

The mutations introduced in Vip3Aa_∆α1, Vip3Aa_41‐KVKK‐44, Vip3Aa_195‐AVAA‐198, Vip3Aa_S‐S, and Vip3Aa_ΔIV‐V (all the engineered mutants except those only changing one residue) were analysed by negative staining transmission electron microscopy to explore if they had an effect at the structural level to influence the switch from protoxin to toxin conformation (Figure 3).

As expected, the WT shows the protoxin conformation before incubation with trypsin and the toxin conformation after incubation with trypsin in a large fraction of the molecules. From our previous structural studies, we know that proteolytic processing is necessary to trigger the toxin conformation, though the percentage of molecules that appear to undergo the conformational change is variable (50%–70%). Interestingly, we observed that the mutant Vip3Aa_∆α1 adopted the toxin conformation independently of the incubation with trypsin. This result indicated that the N‐t comprising helix α1 had a relevant role in the stabilization of the helices at Domain I. Indeed, in the protoxin conformation helix α1 interacts with the core of the tetramer formed by Domain II and it is also connected to the loop α1α2 that allows the twist of helix α2 (Figure 1C). Thus, its absence might release the tension generated by the twist, lowering the energetic barrier required to trigger the remodelling of helices α2 and α3. For the mutants Vip3Aa_41‐KVKK‐44, Vip3Aa_S‐S, and Vip3Aa_ΔIV‐V, the majority of the molecules showed the protoxin conformation before and after incubation with trypsin, which indicates that despite the cleavage is produced, the shift towards the toxin confirmation was not favoured. In the case of Vip3Aa_41‐KVKK‐44, the mutation may have reduced the flexibility of the loop α1α2 in the WT (41‐DTGG‐44) provided by two Gly, slowing down remodelling of the helices. As originally intended, mutant Vip3Aa_S‐S might have cross‐linked helices α2 an α3 by disulphide bonds impairing remodelling. The lack of remodelling of Vip3Aa_ΔIV‐V suggests that the C‐t domains IV and V may provide a frame to sustain Vip3Aa during remodelling. In the case of the mutant Vip3Aa_195‐AVAA‐198, just the protoxin conformation was observed as no cleavage was produced by trypsin.

Thus, our results indicate that remodelling of Domain I is driven by a fine‐tuned mechanism where not only proteolytic cleavage is necessary but also an appropriate free energy barrier to favour the conformational change.

### Insecticidal activity of Vip3Aa and its mutants against *S. exigua* larvae

So far, our structural results indicate that the mutants analysed by EM were not favoured to adopt the toxin conformation in vitro upon trypsin treatment, in contrast to Vip3Aa_∆α1 that could spontaneously adopt the toxin conformation. To test the effect of the mutations on the insecticidal activity of the protein, all the mutants were bioassayed against neonate larvae of the insect species *S. exigua*. The WT protein showed LC_50_ and LC_90_ values of 19.5 (8.6–28) ng/cm^2^ and 62.9 (47–98) ng/cm^2^, respectively (Table 2). The mutant Vip3Aa_Δα1, lacking helix α1, was non‐toxic (Table 2 and Figure S1A); however, the mutant Vip3Aa_41‐KVKK‐44, designed to eliminate the same helix α1 by trypsin digestion, retained toxicity at the LC_50_ level with a value of 55.1 (26–129) ng/cm^2^, whereas it essentially lost all toxicity at the LC_90_ level, indicating a different dose–response compared to the WT protein (Table 2 and Figure S1A). The mutant Vip3Aa_M34L showed enhanced toxicity compared to the WT protein (Table 2 and Figure S1A), with LC_50_ and LC_90_ values of 4.5 (1.2–8.6) and 27.7 (17–46) ng/cm^2^, respectively. The mutant Vip3Aa_195‐AVAA‐198, in which the cleavage site was eliminated, showed toxicity values very similar to that of the WT protein (Table 2 and Figure S1B). In contrast, the mutant Vip3Aa_S‐S, in which the conformational change that follows the proteolytic activation is prevented, showed a complete lack of toxicity (Table 2 and Figure S1B). That lack of toxicity could hardly be attributed to the individual mutations, as the single mutant N69C or the double mutant D66C/N69C showed toxicity values similar to the WT protein (LC_50_ values of 7.5 with FL 4.3–10.5, and 21.0 with FL 15.3–27.4, respectively) and the single mutants Q96C and L100C also showed similar toxicity as the WT (LC_50_ values of 9.6 with FL 6.3–12.8, and 18.4 with FL 6.4–33.3, respectively). The mutants Vip3Aa_L146F and Vip3Aa_L146M, which according to the amino acid substitutions in the atomic models likely count with a reduced internal diameter in the tetrameric coiled‐coil (Figure S5), did not differ from the WT at the LC_50_ level, but had significantly lower toxicity at the LC_90_ level, with a different dose–response compared to the WT protein (Table 2 and Figure S1C). Finally, the mutant Vip3Aa_ΔIV‐V, lacking the C‐t Domains IV and V, also showed a complete lack of toxicity (Table 2 and Figure S1D).

**TABLE 2:** Toxicity of the WT and mutant proteins against *S. exigua* larvae by Probit analysis

Protein	LC_50_ (95% F.L.^a^) ng/cm^2^	Relative potency^b^ (95% F.L.) at LC_50_	LC_90_ (95% F.L.) ng/cm^2^	Relative potency (95% F.L.) at LC_90_	Slope^c^ ± SE^d^	χ^2^ ^e^	DF^f^
Vip3Aa_WT	19.5 (8.6–28)	–	62.9 (47–98)	–	2.5 ± 0.4	45	28
Vip3Aa_∆α1	>315	–	>315	–	–	–	–
Vip3Aa_41‐KVKK‐44	55.1 (26–129)	3.8 (1.9–7.6)	3082 (694–183,530)	53.1 (5.7–498)	0.7 ± 0.2	12	12
Vip3Aa_M34L	4.5 (1.2–8.6)	0.23 (0.18–0.31)	27.7 (17–46)	0.42 (0.28–0.64)	1.6 ± 0.3	34	26
Vip3Aa_195‐AVAA‐198	18.1 (8.8–25)	1.4 (1–2)	56.3 (42–96)	0.9 (0.6–1.3)	2.6 ± 0.6	11	11
Vip3Aa_S‐S	>315	–	>315	–	–	–	–
Vip3Aa_L146F	14.9 (7.9–24)	1.7 (1.1–2.6)	376 (155–2439)	6.3 (2.7–15)	0.9 ± 0.1	24	14
Vip3Aa_L146M	54.4 (17–83)	1.8 (1.2–2.6)	202 (128–485)	3.9 (2–7.5)	2.3 ± 0.6	14	15
Vip3Aa_∆DIV‐V	>315	–	>315	–	–	–	–

^a^ “95% F.L.” indicates the fiducial limits at 95% of confidence.

^b^ Relative potency values around 1 indicate that mutant proteins have similar toxicity compared to the WT, values higher than 1 indicate lower toxicity for the mutants, whereas relative potency values smaller than 1 indicate enhanced toxicity.

^c^ Similar slope values indicate similar dose‐responses.

^d^ SE indicates the standard error for the calculated slope values.

^e^ Higher χ^2^ values indicate higher heterogeneity of the data in the Probit analysis.

^f^ DF indicates degrees of freedom for the calculation of χ^2^.

Overall, these results indicate that helix α1 is essential for Vip3Aa activity, as well as remodelling of helices α2 and α3 and the presence of the C‐t domains. The increased activity of Vip3Aa_M34L reinforces the role of helix α1 as a hot spot in the mode of action. Moreover, Vip3Aa_41‐KVKK‐44 has a decreased activity, as Vip3Aa_L146F and Vip3Aa_L146M. The fact that Vip3Aa_195‐AVAA‐198 showed the same insecticidal activity as the WT suggests an alternative in vivo cleavage site.

### Proteolytic processing of the Vip3Aa_195‐AVAA‐198 mutant protein

**FIGURE 4:**
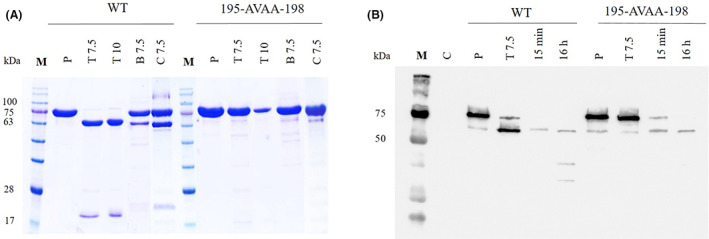
Proteolytic processing of the Vip3Aa_195‐AVAA‐198 mutant under different conditions. (A) The proteins were incubated in vitro under different conditions and then subjected to SDS‐PAGE. P, protoxin samples; T7.5, samples digested with trypsin (10% w/w) at pH 7.5; T10, samples digested with trypsin (10% w/w) at pH 10; B7.5, samples digested with *S. exigua* BBMV (10% w/w) at pH 7.5; C7.5, samples digested with α‐chymotrypsin (10% w/w) at pH 7.5. Band sizes were estimated according to the Blue Star molecular marker (M) (NIPPON Genetics). (B) Larvae fed with either protein were dissected after 15 min or 16 h and the midgut sample was subjected to SDS‐PAGE. The Vip3Aa (WT and mutant) digestion fragments were detected by Western blot. C, control midgut sample from non‐intoxicated larvae; P, controls of protoxin samples (100 ng); T7.5, controls of in vitro trypsin‐treated proteins at pH 7.5 (100 ng); 15 min, in vivo processed proteins after 15 min of ingestion; 16 h, in vivo processed proteins after 16 h of ingestion. Band sizes were estimated according to the Dual Color molecular marker (M) (NIPPON Genetics).

The observation that the mutant Vip3Aa_195‐AVAA‐198, engineered to destroy the cleavage site, showed similar toxicity values compared to the WT despite the fact that it was not activated in vitro by commercial trypsin and only barely by *Se*MJ, made us search for other in vitro conditions that emulated better the in vivo conditions. Thus, we performed the in vitro digestion at higher pH, incubating with *S. exigua* BBMV, and replacing trypsin with α‐chymotrypsin, another relatively common serine peptidase in the lepidopteran larvae midgut (Caccia et al., 2014) (Figure 4A). The mutant protein could not be processed under any of these new conditions, whereas the WT protein was completely processed by trypsin at both pH conditions and partially processed by *S. exigua* BBMV and α‐chymotrypsin.

Since we failed to show the in vitro processing of the Vip3Aa_195‐AVAA‐198 mutant, we set out to demonstrate that this protein was processed at an alternative cleavage site in vivo. The in vivo digestion assay was performed by drop‐feeding *S. exigua* larvae with either the mutant or the WT protein and then dissecting the larvae at different times. The processed proteins were detected by Western blot (Figure 4B). The results showed that both, the WT and mutant Vip3Aa_195‐AVAA‐198 were completely processed in vivo after 16 h of ingestion. However, the kinetics of activation for the mutant protein seemed to be slower, since after 15 min of ingestion the WT protein was completely processed whereas almost half of the mutant proteins still remained in the protoxin form. The in silico analysis of the proteolytic cleavage sites in the surroundings of 195‐AVAA‐198, using the ExPASy Peptide Cutter tool (Bioinformatics Resources, Swiss Institute of Bioinformatics), predicts 14 potential trypsin/chymotrypsin cleavage sites in a range from 8 to 22 amino acids upstream and downstream of the 195‐AVAA‐198 sequence. Most probably, in vivo, some of these sites become available for trypsin or chymotrypsin cleavage.

## DISCUSSION

The recent structures of Vip3Aa and Vip3B in the protoxin and toxin conformation have revealed that remodelling events at Domain I may impact insecticidal activity. To shed light on this mechanism, we engineered several Vip3Aa mutants, in highly conserved residues of Domain I (Figure S6), and then, characterized their proteolytic and insecticidal activity, as well as their conformational flexibility trying to correlate their structural features with their activity in vivo.

Our data indicate that remodelling and toxicity are interconnected by a fine‐tuned mechanism where the free energy that involves remodelling depends on the appropriate coordination of several structural features. One of these structural features corresponds to helix α1 which has proven to be essential. Its absence leads to a complete loss of toxicity, in agreement with previous reports showing that the lack of the first N‐t 33 and 39 residues abolished the insecticidal activity of the protein (Bhalla et al., 2005; Selvapandiyan et al., 2001). Our data also reveal that the absence of helix α1 induces the spontaneous conformation of toxins. Thus, we can envision that helix α1 may be holding Domain I in the protoxin conformation through interactions with Domain II until the protein has been proteolytically processed in the surroundings of the membrane of the midgut cells, to favour remodelling and interaction with the membrane at the appropriate location and timing. The mutant Vip3Aa_M34L, which carries a point modification inside helix α1, shows enhanced toxicity compared to the WT protein, in agreement with previous results obtained with the Vip3Af protein and the related species *Spodoptera littoralis* (Banyuls et al., 2021). The fact that a point mutation inside this region increases the toxicity can be related to the amphipathic nature of helix α1, increasing its hydrophobicity to improve the interaction with the hydrophobic nature of the membrane since the hydrophobic index of Leu (0.943) is higher than Met (0.738) (Black & Mould, 1991). In amphipathic helices (AH), the hydrophobic side of the helix inserts into the interior of the bilayer, while the hydrophilic side interacts with the lipid headgroups (Drin & Antonny, 2010; Roberts et al., 2013), thus, promoting membrane curvature and membrane fission (Campelo et al., 2008; Martyna et al., 2017). AHs of many fission‐inducing proteins contain at least two basic residues (Arg and/or Lys) each at the polar‐non‐polar interface (Zhukovsky et al., 2019). In helix α1, its sequence 23‐IYGFATGIKDIMNMIFK‐39 contains two Lys, K31 and K39 which may indicate that Vip3Aa could induce membrane fission. Then, that would lead to a better insertion in the membrane to further interact with some transmembrane or intracellular compounds to lead the cell death, possibly through its internalization and activation of the apoptotic pathways as has already been described (Hernández‐Martínez et al., 2017; Hou et al., 2020; Jiang et al., 2016; Nimsanor et al., 2020).

Vip3 proteins can get inserted into the membrane of liposomes through the N‐t part of Domain I (Byrne et al., 2021) and have been described as pore‐forming proteins (Lee et al., 2003; Liu et al., 2011). The tetrameric coiled‐coil in the structure of Vip3Aa holds a divalent ion bound in an internal diameter of ~3 Å (Núñez‐Ramírez et al., 2020) which size could impact pore formation, although it is much narrower compared to the pore diameters described for other pore‐forming proteins (Dal Peraro & van der Goot, 2016; Meusch et al., 2014; Mueller et al., 2009). Thus, mutations at L146 to Phe and Met were aimed to reduce the diameter and possibly affect ion transportation, as these are more voluminous residues than Leu. However, just a reduction of toxicity at the LC_90_ level was detected, indicating that the inside diameter of the coiled‐coil is not a critical feature for Vip3Aa insecticidal activity. Thus, ion binding to the tetrameric coiled‐coil might help to maintain the structural stability and dynamics of the coiled‐coil (Hartmann, 2017) and not serve for ion transportation, though it cannot be ruled out that the interaction of Domain I with the membrane might facilitate that ions cross the membrane through other mechanisms.

A second structural feature that is essential for the Vip3Aa activity is the remodelling of helices α2 and α3, as the mutant Vip3Aa_S‐S which locked the protein in the protoxin conformation impaired the insecticidal activity. These two long helices comprise the total length of the tetrameric coiled‐coil (Figure 1B) possibly serve as a scaffold for the action of helix α1 in the activity of the toxin.

A third structural feature that has been demonstrated to be relevant is the loop α1α2 (residues 40–47). We mutated the sequence 41‐DTGG‐44, which contains two Gly, for 41‐KVKK‐44, resulting in the reduction of the toxicity of this mutant. Although our mutation was intended to introduce a cleavage site for the release of helix α1, the mutation has demonstrated that stiffness in the loop between helices α1 and α2 is deleterious. Thus, flexibility in this region is important for remodelling and therefore, for the insecticidal activity of the protein.

A fourth structural feature that unexpectedly has resulted to be critical for remodelling is the C‐t part of the protein, formed by Domains IV and V which are not interacting with Domain I. Their absence in Vip3Aa favoured the protoxin conformation and abrogated toxicity. This is in agreement with previous reports showing a drastic lack of toxicity of C‐t truncated Vip3Aa in bioassays with several lepidopteran pests, including *S. exigua* (Banyuls et al., 2018, 2021; Gayen et al., 2012, 2015; Li et al., 2007; Quan & Ferré, 2019; Selvapandiyan et al., 2001). The fact that the protoxin conformation was favoured in the absence of Domains IV and V points out a role for the C‐t during remodelling, most likely through the interaction with the epithelial membrane of the cells. Thus, the absence of both domains may impair Vip3A from targeting the membrane, affecting recognition to find an interacting surface. However, we cannot discard that the lack of these domains could affect the proteolytic activation process of the protein in vivo, and so, the absence of toxicity could be the result of different factors.

Finally, the 195‐AVAA‐198 cleavage site is also an essential structural feature for remodelling and toxicity. Surprisingly, we have found that alternative proteolytic processing may also take place in vivo, and this explains why the toxicity of this mutant does not differ from that of the WT, at least against *S. exigua*. Our results are in contrast to those obtained by Zhang et al. (2018), where the same mutant protein showed a significant reduction of toxicity to *S. exigua*. It is important to note that the mortality values obtained by these authors for all the Vip3Aa proteins tested were much lower than ours, suggesting important differences in the susceptibility of the *S. exigua* colonies against Vip3Aa proteins.

In conclusion, we have demonstrated here the crucial role of helix α1and the remodelling which takes place in the toxin conformation at Domain I for the insecticidal activity of the Vip3Aa protein. We have proposed several structural features that are relevant for the timing in which Vip3Aa remodelling occurs to drive its biological activity: the role of helix α1, essential for toxicity, holding the Domain I in the protoxin conformation and most probably interacting with the membrane in the toxin conformation, the flexibility of loop α1α2, the remodelling of helices α2 and α3, the proteolytic processing that takes place at the loop α4α5 between Domains I and II and the role of the C‐t Domains IV and V to sustain the conformational change.

## AUTHOR CONTRIBUTIONS

ML‐B, YB, PC, and JF designed the experiments. ML‐B, FP‐M, RN‐R, and YB performed the experimental work. ML‐B, YB, EA‐P, PC, and JF discussed and interpreted the results. ML‐B, PC, and JF wrote the main manuscript text. ML‐B, EA‐P, and PC prepared the figures. All authors reviewed the manuscript.

## CONFLICT OF INTEREST

The authors declare no competing interests.

## Supporting information


Appendix S1

## Data Availability

The authors confirm that the data supporting the findings of this study are available within the article and supporting information.
