# Supplementary material for: Structural and functional role of Domain I for the insecticidal activity of the Vip3Aa protein from Bacillus thuringiensis
Source: Microb Biotechnol. 2022 Jul 13;15(10):2607–18. doi: 10.1111/1751-7915.14110 (PMC9518980; doi:10.1111/1751-7915.14110)
Supplement: Supplementary file 1 — Appendix S1 [file MBT2-15-2607-s001.docx]

**SUPPORTING INFORMATION**

Structural and functional role of Domain I for the insecticidal activity of the Vip3Aa protein from *Bacillus thuringiensis*

Maria Lázaro-Berenguer^1,2^, Francisco Paredes-Martínez^2,3^, Yolanda Bel^1,2^, Rafael Núñez-Ramírez^4^, Ernesto Arias-Palomo^4^, Patricia Casino^2,3,5^*, Juan Ferré^1,2,^*

^1^ Department of Genetics, Universitat de València, Dr. Moliner 50, 46100 Burjassot, Spain

^2^ Institut Universitari de Biotecnologia i Biomedicina BIOTECMED, Universitat de València, Dr. Moliner 50, 46100 Burjassot, Spain

^3^ Department of Biochemistry and Molecular Biology, Universitat de València, Dr. Moliner 50, 46100 Burjassot, Spain

^4^ Centro de Investigaciones Biológicas Margarita Salas, CSIC, 28040 Madrid, Spain

^5^ CIBER de Enfermedades Raras (CIBERER-ISCIII), Madrid, Spain.

*Corresponding Author: Juan Ferré, e-mail: [juan.ferre@uv.es](mailto:juan.ferre@uv.es)

*Corresponding Author: Patricia Casino, e-mail: patricia.casino@uv.es

Running Title: Structural and functional role of Vip3Aa Domain I


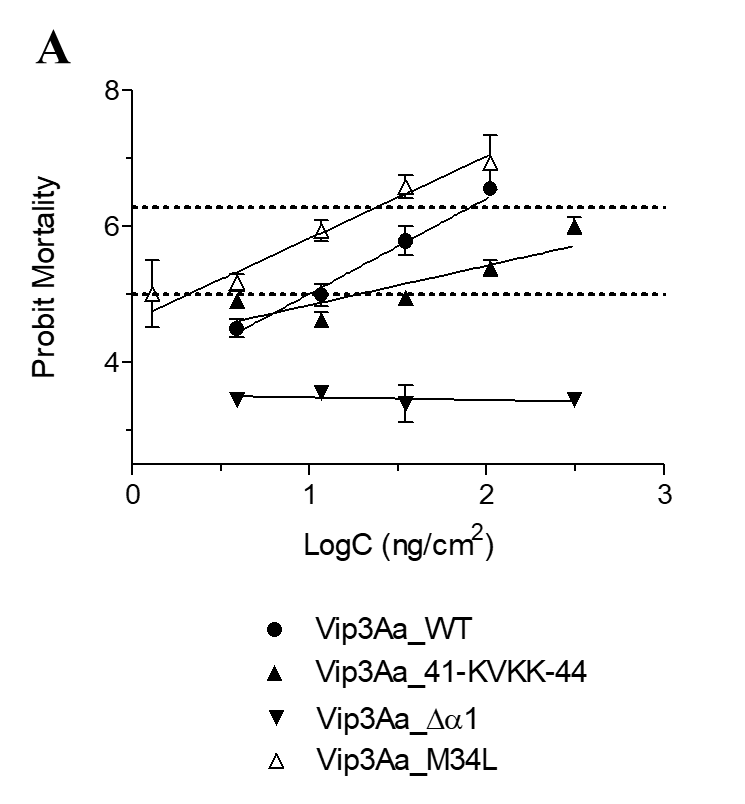

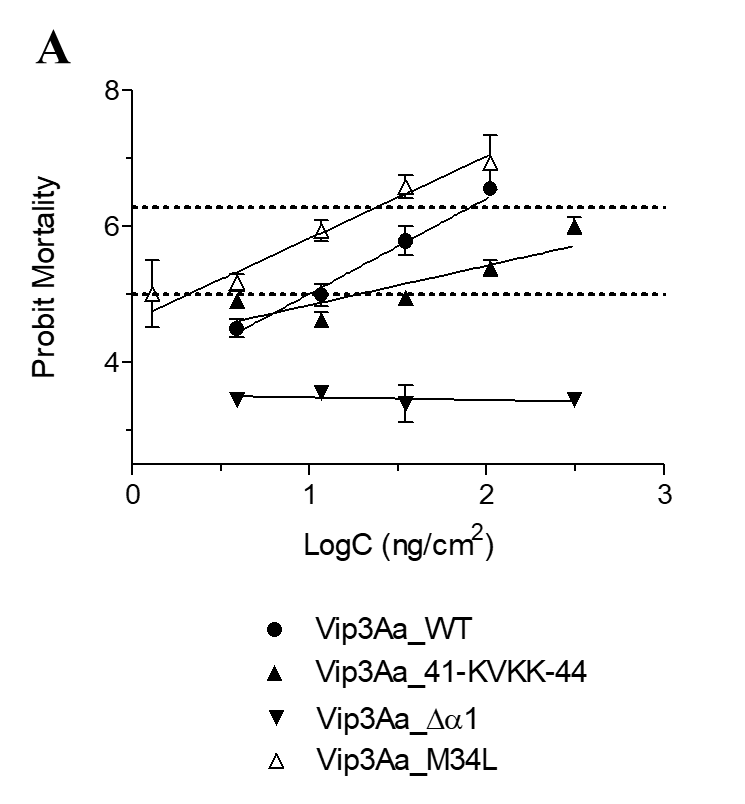

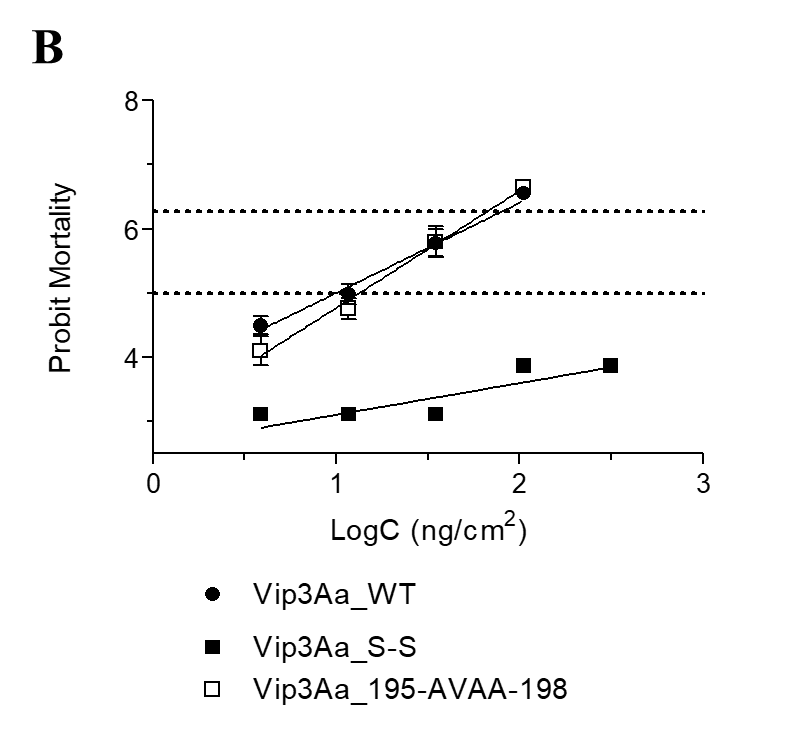

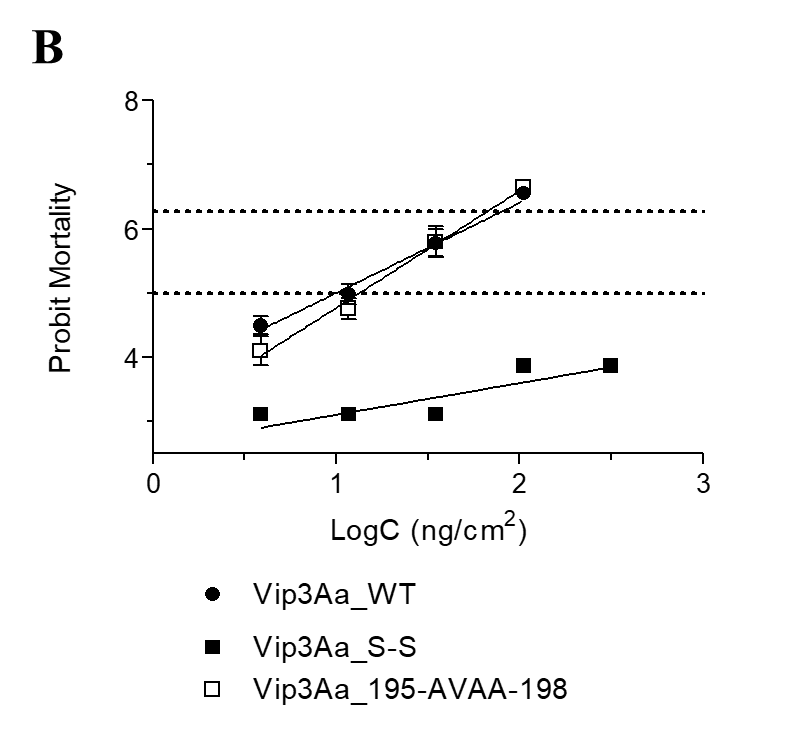

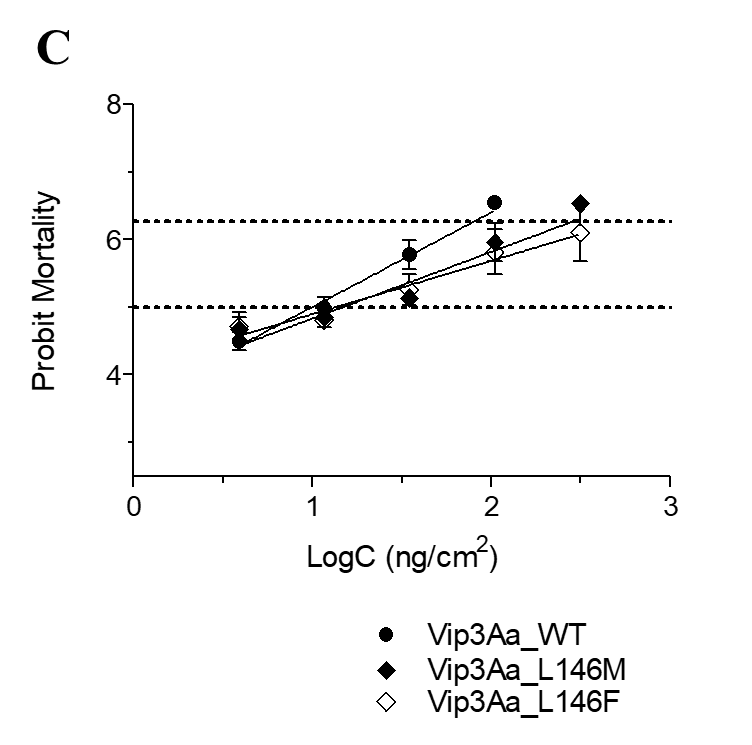

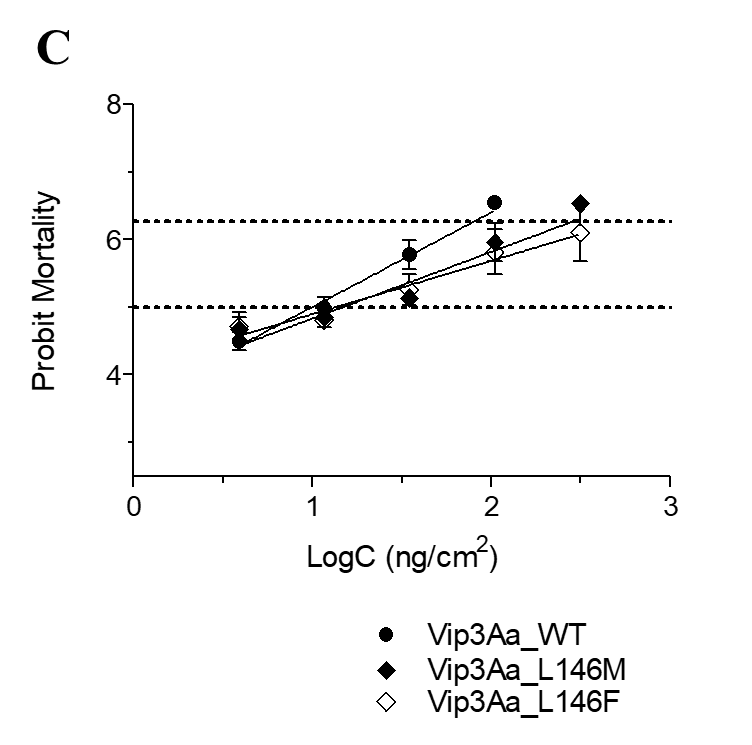

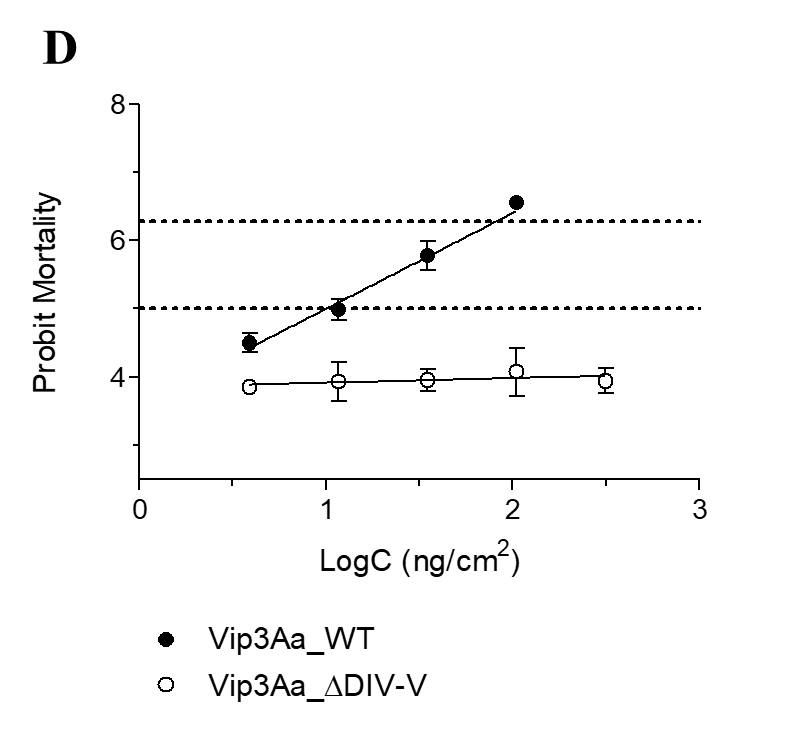

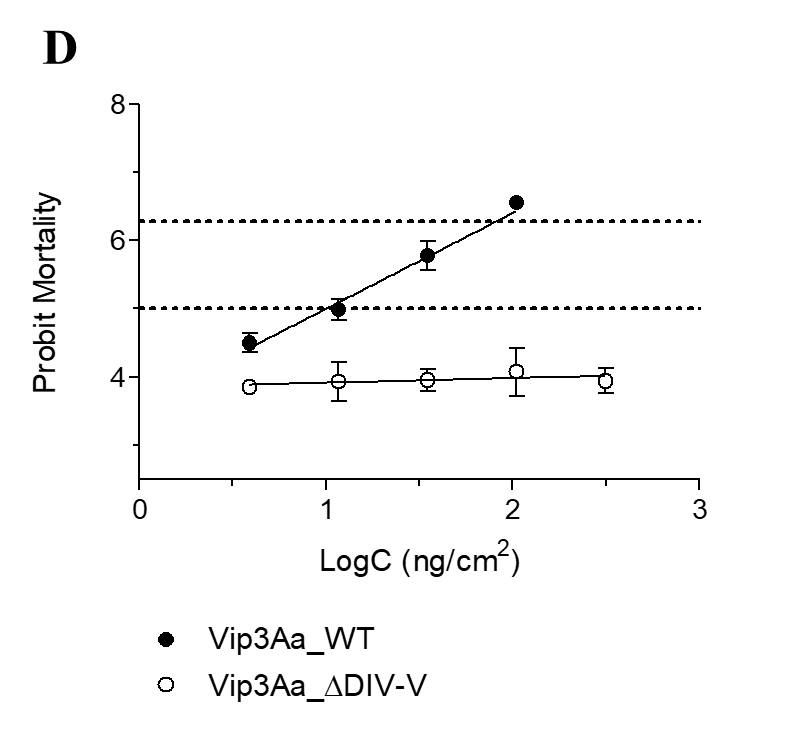


**Supplementary Figure S1. Dose-response of the Vip3Aa WT and mutant proteins against *S. exigua* larvae.** Panel A includes the mutants related to the modification of the N-t helix α1. Panel B includes the mutants related to the impairment of the trypsin processing and Domain I remodelling. Panel C includes the mutants related to the reduction of the inside diameter of the coiled-coil that Domain I forms in the activated conformation. Panel D shows the mutant lacking the C-t domains IV and V. Dashed lines indicate 50% and 90% mortality in the Probit scale.


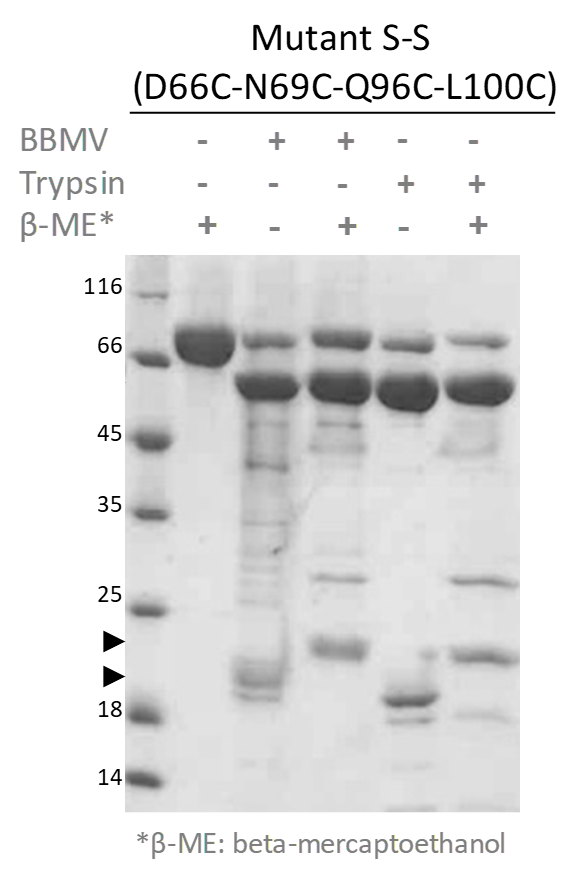


**Supplementary Figure S2. Electrophoretic mobility of Vip3_S-S mutant in SDS-PAGE.** Proteolytic processing in the presence of BBMV or trypsin together with the absence and presence of the reducing agent β-mercaptoethanol. Arrowheads indicate the bands corresponding to Domain I.

**Supplementary Figure S3. MALDI-TOF analysis of Vip3Aa S-S N-terminal fragments.** The analysis was carried out in the absence (sample 1) or the presence of the reducing agent β-ME (sample 3) obtained after trypsin digestion. Samples were excised from the gel upon usage. Peptides containing free Cys were found just in the reduced sample 3 for the peptides shown in the spectra.

**Supplementary Figure S4. LC-MS analysis of Vip3Aa S-S N-terminal fragments.** The analysis was carried out in the absence (sample 1) or the presence of the reducing agent β-ME (sample 3) obtained after trypsin digestion. Samples were excised from the gel and processed for mass spectrometry analysis. Extracted ion chromatograms (XIC) of the peptides indicated in the table are shown. Chromatograms from sample 1 are shown in black whereas those from sample 3 are shown in red. The identity of the peptides was confirmed by tandem mass spectrometry.

**Supplementary Figure S5. Modelled mutations in the toxin structure of Vip3Aa (PDB: 6TFK) of L146 to Phe and Met.** Minimization steps have been conducted after the mutations have been modelled using Chimera UCSF, then, distances between side chains are shown.

**Supplementary Figure S6. Multiple sequence alignment of Vip3 family members showing conservation of residues mutated.** The protein sequences used are as follows: Vip3Aa1 (GenBank accession number Vip3Aa (AAC37036), Vip3Ab1 (AAR40284), Vip3Ad2 (CAI43276), Vip3Ae1 (CAI43277), Vip3Af1 (CAI43275), Vip3Ag2 (ACL97352), Vip3Ah1 (ABH10614), Vip3Ai1 (KC156693), Vip3Aj1 (KF826717), Vip3Ba1 (AAV70653), Vip3Bb2 (ABO30520), and Vip3Ca1 (ADZ46178).

**Supplementary Table S1. Primers used for mutagenesis**

| **Vip3Aa_mutant (residues)** | **5’ → 3’ primer sequence** |
| --- | --- |
| **∆α1** (residues 40-789) | CAGGGACCCGGTACGGATACAGGTGGTGATCTAAC  CGAGGAGAAGCCCGGTTACTTAATAGAGACATCGTAAAAATGTAC |
| **S-S** (residues 10-789)  1^st^ Double mutation  D66C-N69C  D66C-N69C  2^nd^ Double mutation over 1^st^  Q96C-L100C  Q96C-L100C | GTGTGCGGAAGCTTAAATGATCTTATC  CCCGCACAATTTACCAGAAATATCATTTAG  AGTTTGCAATGATGTTAATAACAAACTCG  TGATTGCATTCATTTGCAATTTTTAATATTTCC |
| **195-AVAA-198** (residues 10-789) | GCGGCGGATGGCTCTCCTGCAGAT  TACCGCTGAACTAGTTTCTGTAGCAAAAG |
| **41-KVKK-44** (residues 10-789) | AAAAAGGATCTAACCCTAGACGAAATTTTAAAG  TACTTTCGTTTTAAAAATCATGTTCATAATGTC |
| **L146M** (residues 10-789) | AAGTAAACAAATGCAAGAGATTTC  AAGTATTCTATTTGCAGACTTAG |
| **L146F** (residues 10-789) | AAGTAAACAATTCCAAGAGATTTCTG  AAGTATTCTATTTGCAGACTTAG |
| **∆IV-V** (residues 10-532) | CAGGGACCCGGTACAAGAGCCTTACCAAGTTTTATTG  CGAGGAGAAGCCCGGTTAACTTGGCGGGACGATCAATTTAG |
| **M34L** (residues 1-789)  Mutation M34L | AGACATTTTGAACATGATTTTTAAAACGGATACAGGTG  TCATGTTCAAAATGTCTTTGATACCAGTGGC |
